# Clinical and healthcare research

**DOI:** 10.1007/s00068-025-02859-x

**Published:** 2025-05-06

**Authors:** Philipp Störmann, René Verboket, Ingo Marzi

**Affiliations:** Department of Trauma Surgery and Orthopedics, University Hospital, Goethe University Frankfurt, Frankfurt am Main, Germany

**Keywords:** WhiteBook, Polytrauma, ESTES

## Abstract

Research in trauma care is indispensable for advancing the field of trauma surgery and healthcare delivery. The outcomes of such research have the potential to save lives, reduce disability, and optimise healthcare resource allocation. This chapters summarises key types of research in trauma care, highlights funding opportunities, and outlines future research priorities in Europe.

## Introduction

Clinical research plays a pivotal role in advancing trauma surgery and improving healthcare outcomes. Trauma surgery involves managing critically injured patients, often in life-threatening scenarios requiring rapid, evidence-based interventions. Clinical research serves as the foundation for improving patient outcomes, refining treatment strategies, and optimising the delivery of trauma care.

Translational research bridges the gap between experimental studies and clinical practice, enabling the implementation of innovative procedures, agents, or medications that have demonstrated promise in preclinical settings. However, introducing new concepts into patient care involves significant administrative and regulatory challenges.

Healthcare research in trauma surgery is inherently multidisciplinary, addressing a wide range of complex issues, including optimal resuscitation protocols, surgical techniques, postoperative care, and long-term rehabilitation. Through systematic investigations, researchers evaluate the safety, efficacy, and applicability of new interventions, while identifying areas for improvement in existing practices.

Clinical research underpins evidence-based decision-making, ensuring that trauma care aligns with the latest knowledge and best practices. This encompasses health services research, which analyses large-scale patient databases, such as insurance data and quality registries, to assess healthcare delivery and outcomes. By fostering rigorous scientific inquiry, this research drives advancements that save lives, reduce disability, and enhance healthcare resource efficiency, ultimately improving patient well-being and recovery.

## Basic research

Basic research in trauma surgery forms the cornerstone of the trauma research continuum, providing critical insights into the cellular, molecular, and physiological processes underlying traumatic injuries. These findings serve as the foundation for translational and clinical innovations aimed at improving trauma care.

Key areas of basic research in trauma surgery:

- Injury mechanisms:

Basic research focuses on elucidating the complex mechanisms by which traumatic injuries occur, including tissue damage, inflammation, coagulation cascades, and cellular responses. Understanding these processes is essential for guiding clinical decision-making and developing effective interventions.

- Wound and bone healing and regeneration

Investigating the biology of wound healing, bone repair, and tissue regeneration is crucial for trauma surgery. Research explores factors influencing tissue repair, scar formation, and regenerative potential, informing the development of interventions to optimise healing and recovery.

- Biomarker discovery

Biomarkers are critical for assessing injury severity, predicting prognosis, and monitoring treatment response. Basic research aims to identify and validate biomarkers that enable clinicians to make timely, evidence-based decisions. The future focus will be on understanding the source, function, and clinical significance of these biomarkers to enhance patient care.

- Drug development

Basic research provides the foundation for developing pharmacological agents that address key challenges in trauma care, such as inflammation, coagulation, pain management, and infection control. A future goal is personalised pharmacotherapy, tailored to the individual patient’s pathophysiological profile for optimal outcomes.

- Biomechanics

Biomechanical research investigates the forces and stresses that lead to different types of injuries, providing valuable insights for designing protective equipment and safety measures to prevent trauma. Additionally, biomechanical considerations are critical for developing effective implants, prosthetics, and biological scaffolds.

## The role of basic research

Basic research is defined by its commitment to uncovering fundamental principles and expanding our understanding of traumatic injuries. While the immediate clinical applications may not always be apparent, the knowledge gained from basic research is indispensable for driving advancements in trauma care.

By providing essential insights into injury mechanisms, healing processes, and therapeutic targets, basic research fuels translational and clinical studies that lead to tangible improvements in patient outcomes. This relentless pursuit of knowledge ultimately enhances trauma care, reduces morbidity, and contributes to better long-term recovery for trauma patients.

## Translational research

Translational research in trauma surgery serves as a vital bridge between laboratory discoveries and clinical practice, aiming to improve patient outcomes and advance the field of trauma care. This multidisciplinary approach transforms insights from basic science into practical applications that address real-world challenges in trauma management. It is a dynamic process that continuously refines scientific knowledge to deliver tangible benefits to patients.

Key objectives of translational research in trauma surgery:

- Bridging bench to bedside

Translational research begins with understanding injury mechanisms, wound healing, and tissue regeneration through laboratory studies. Researchers identify novel therapeutic targets, biomarkers, and innovative strategies, which are then rigorously evaluated and integrated into clinical practice.

- Improving diagnostics

Enhancing the speed and accuracy of trauma diagnosis is a central goal. Translational research drives the development of advanced imaging techniques, biomarker-based diagnostics, and point-of-care tools, enabling clinicians to assess injury severity and initiate timely interventions.

- Innovating surgical techniques

Translational efforts fuel the creation of advanced surgical approaches, including minimally invasive techniques and robotic-assisted surgeries. These innovations aim to optimise patient outcomes while minimising surgical trauma and recovery times.

- Personalised medicine

Tailoring treatment to individual patients based on genetic profiles, injury-specific characteristics, and anatomical variations is a cornerstone of translational research. By integrating patient-specific data, clinicians can make more precise therapeutic decisions, improving recovery rates and outcomes.

- Rehabilitation, long-term care, and quality of life

Translational research extends beyond immediate surgical care, focusing on innovative rehabilitation strategies and long-term patient management to enhance functional recovery. Measuring quality of life outcomes is crucial for evaluating the effectiveness of therapeutic and surgical interventions.

Translational research operates as a continuum, where findings are validated through clinical trials, refined based on patient outcomes, and iteratively improved. This dynamic process allows trauma surgeons to integrate the latest advancements into clinical practice, enhancing survival rates, reducing disability, and improving patients'overall quality of life. The collaborative efforts of researchers, clinicians, and healthcare providers underpin the evolution of trauma surgery, ensuring that innovations translate into meaningful benefits for trauma patients.

## Clinical research

Clinical research in trauma surgery is essential for advancing treatment protocols, improving patient outcomes, and refining surgical techniques. Unlike isolated case reports or single-centre studies, valid clinical research requires well-designed, prospective trials with predefined hypotheses and sufficient statistical power. If patient recruitment is limited, multicentre studies should be prioritised to ensure robust and generalisable findings.

Compared to registry studies, clinical trials allow researchers to investigate causal relationships, answering targeted research questions with greater precision. Establishing multicentre and multinational trials is critical for achieving the highest-quality results and advancing evidence-based practices in trauma surgery.

Key contributions of clinical research in trauma surgery

- Optimising treatment strategies

Clinical research identifies the most effective practices for managing trauma cases, considering factors such as injury severity, patient demographics, and recovery trajectories. Through rigorous trials and longitudinal studies, researchers evaluate the optimal timing, techniques, and adjunct therapies to maximise recovery and minimise long-term complications.

- Adopting innovative technologies

Clinical research facilitates the safe evaluation and adoption of emerging technologies, tools, and surgical techniques. By assessing the efficacy, safety, and benefits of innovations such as advanced implants, robotic-assisted surgery, and novel devices, researchers ensure that trauma surgeons have access to cutting-edge tools for improved precision, reduced operative times, and fewer postoperative complications.

- Promoting collaboration

Clinical research fosters the exchange of knowledge and expertise among healthcare professionals, driving interdisciplinary collaboration. This shared understanding accelerates the dissemination of best practices and enhances the collective ability to address the complexities of trauma care.

- Public funding and research prioritisation

To advance trauma care, robust public funding support is essential for well-defined clinical studies. By prioritising and funding high-quality research initiatives, public agencies can drive transformative breakthroughs that optimise treatment strategies, improve patient outcomes, and enhance long-term quality of life for trauma survivors.

Through meticulously designed trials, clinical research serves as a catalyst for continuous improvement in trauma care. It ensures that treatment strategies are evidence-based, innovations are rigorously evaluated, and patients receive the best possible care. By fostering collaboration and securing adequate funding, clinical research will continue to shape the future of trauma surgery and deliver meaningful advancements for patients and healthcare systems alike.

## Registry research

Trauma registries are well established in many European countries, including the Trauma Register of the German Society for Trauma Surgery, the Trauma Audit and Research Network (TARN) of the British NHS, and registries in Scandinavia, the Netherlands, and other regions. These registries typically follow defined datasets, enabling system comparisons using frameworks such as the Utstein template.

The primary strength of registry research is its ability to reflect the realities of clinical care through broad datasets and large patient numbers, which often provide results with high external validity. Such data are frequently used for quality assurance, allowing evaluation of care structures, procedural processes, and the effects of new measures or changing conditions on patient outcomes (Q: Weißbuch 3.0).

To ensure meaningful and clinically relevant conclusions, registry data must meet several criteria:

- Data quality

High-quality, complete datasets are essential. Centralised monitoring systems, including plausibility filters, can significantly improve data accuracy and reliability. (Q: Bouillon et al., Unfallchirurg 2016 119:469–474).

- Clear definitions

Precise definitions of individual data points are needed to ensure accuracy and clinical relevance.

- Statistical relevance

Larger registries increase the risk of detecting statistically significant but clinically irrelevant results. Therefore, research questions must be clearly defined a priori to maintain focus and validity

- Correlations vs. causality

It is critical to recognise that registries identify correlations, not causality, when interpreting results.

Sustainable funding and active participation by healthcare providers are key to maintaining robust trauma registries. Motivating centres to consistently enter data ensures completeness and reliability, allowing registries to drive meaningful advancements in trauma care.

## Health services research

Health services research examines the organisation, management, and financing of trauma care. Closely linked to registry-based research, it provides insights into the practical implementation of new clinical measures.

This research often utilises large datasets from hospitals, insurance providers, and national healthcare systems to assess established and newly introduced treatment protocols over extended follow-up periods. By identifying trends and early indicators, health services research highlights areas for improvement and supports further clinical investigations.

The findings of health services research directly influence healthcare policy and funding decisions, guiding resource allocation, care structures, and the overall financing of trauma care systems. These results are particularly valuable to governments and policymakers striving to optimise cost-efficiency and improve patient outcomes.

## Prevention of trauma

Trauma prevention is of paramount importance to communities, insurers, and policymakers. Research in this area demonstrates how technical advances, public policies, and awareness campaigns can significantly reduce the incidence of severe trauma and its associated long-term costs.

A notable example is the improvement in car safety over recent decades, which has effectively prevented severe injuries and reduced follow-up care costs for individuals and insurers alike. Similarly, workplace safety initiatives, led by worker compensation organisations and government bodies, have successfully limited occupational injuries, benefiting both individuals and insurers.

However, evolving societal trends introduce new challenges. For instance, the growing popularity of cycling and e-scooter use has led to a surge in related injuries, undermining existing prevention strategies. Trauma prevention research must adapt to changing patterns, offering innovative solutions to mitigate risks and protect public health.

## Public interest and funding

Severe trauma has profound consequences for patients, their families, and society. Traumatised individuals often face sudden disruptions to their personal and professional lives, including loss of income, career opportunities, and social stability. These challenges are further exacerbated by financial strain, particularly for patients without robust insurance or social support systems.

Compared to patients with cardiovascular diseases or cancers, trauma patients are often younger and of working age. The societal impact of trauma is therefore disproportionately high, as it results in lost productivity and increased reliance on insurance and pension systems. Given these far-reaching implications, trauma prevention and research warrant significant public and private interest.

## Funding opportunities

Trauma research is supported through diverse funding channels, including national, European, and transnational grants. Examples include:

**Table:** 

Name	Country/region	Funding options	Internet
European Research Council (ERC)	Europe	Any research; EU membership required	https://erc.europa.eu/
European Society of Trauma and Emergency Surgery (ESTES)	Europe	Scholarships and small grants	https://www.estesonline.org
German Scientific Society (DFG)	Germany	Basic and clinical research	https://www.dfg.de/ https://anr.fr/en/
French National Research Agency (ANR)	France	General research initiatives	
Osteosynthesis & Trauma Care Foundation (OTC)	Europe	Research grants up to $50,000 for members	https://otcfoundation.org/research/
AO Trauma Foundation	Europe	Mini and large project grants for members	https://www.aofoundation.org/
Horizon Europe	Europe	EU research and innovation funding	https://research-and-innovation.ec.europa.eu/

These opportunities highlight the breadth of support available for trauma research, encouraging researchers to pursue innovative projects that address key challenges in trauma care.

## Conclusion and needs for the future

The future of trauma research—encompassing translational, clinical, and health services research—relies on addressing complex healthcare challenges, integrating advanced technologies, and fostering interdisciplinary collaboration. Key priorities include:

- *Defining optimal trauma care in Europe*: Establishing benchmarks for high-quality trauma care that accommodate the diversity of European healthcare systems.
- *Data integration and standardisation:* Developing a unified European trauma registry, funded by the EU, to enable seamless data collection, integration, and analysis across systems.
- *Facilitating clinical trials:* Streamlining regulatory processes to facilitate large-scale, multicentre clinical studies that introduce safer, more effective treatment procedures. Cross-border collaboration and investment in research infrastructure are critical to achieving this goal.
- *Prioritising prevention research:* Supporting studies that focus on injury prevention, workplace safety, and early intervention strategies to mitigate long-term trauma impacts.
- *Advancing translational research:* Allocating resources to innovative translational research that bridges scientific discovery and clinical practice. Prioritising personalised medicine, novel diagnostics, and rehabilitation strategies will enhance patient-centred care.

By addressing these priorities, trauma research can deliver transformative advancements, improving survival rates, reducing disability, and enhancing the quality of life for trauma patients. Sustained public and private funding, along with cross-disciplinary collaboration, will be essential for driving progress and meeting the evolving challenges of trauma care.

## Data Availability

No datasets were generated or analysed during the current study.
